# Supplementary material for: Efficient search, mapping, and optimization of multi-protein genetic systems in diverse bacteria
Source: Mol Syst Biol. 2014 Jul 1;10(6):731. doi: 10.15252/msb.20134955 (PMC4265053; doi:10.15252/msb.20134955)
Supplement: Supplementary file 1 — Supplementary Figure S1 [file msb0010-0731-sd1.pdf]

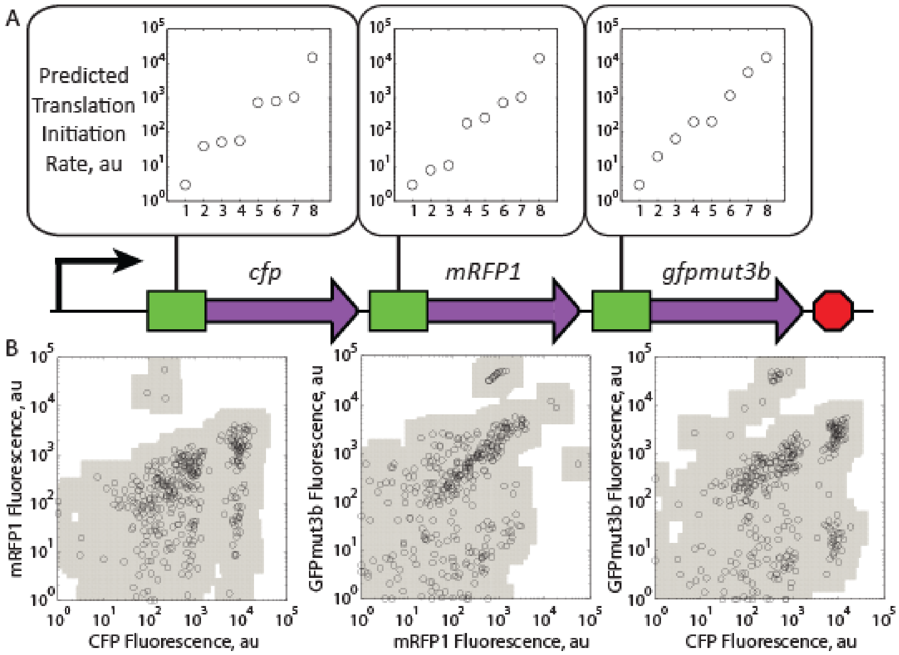

Supplementary Figure S1: Sampling 3-dimensional CFP, mRFP1, GFPmut3b production rate space using optimized RBS libraries. (A) Three optimized RBS libraries were designed using the RBS Library Calculator (search resolution=0.35) to contain 8 variants that efficiently search the translation rate space of a 3-color operon encoding CFP, mRFP1, and GFPmut3b (**Supplementary Table 4**). A combinatorial library of three colored operon was created from these RBS libraries and transformed into *E. coli*. (B) Five hundred colonies were randomly selected and characterized for their fluorescence production.
