# Supplementary material for: Efficient search, mapping, and optimization of multi-protein genetic systems in diverse bacteria
Source: Mol Syst Biol. 2014 Jul 1;10(6):731. doi: 10.15252/msb.20134955 (PMC4265053; doi:10.15252/msb.20134955)
Supplement: Supplementary file 2 — Supplementary Figure S2 [file msb0010-0731-sd2.pdf]

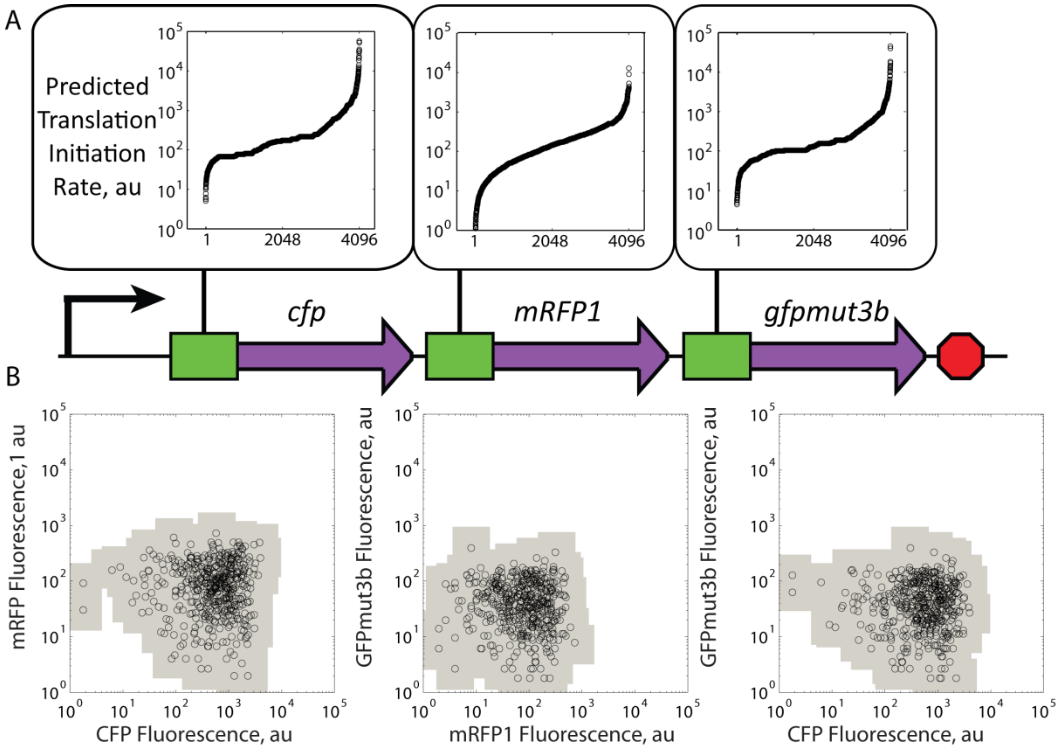

Supplementary Figure S2: Sampling 3-dimensional CFP, mRFP1, GFPmut3b production rate space using optimized RBS libraries. (A) A library of triple-color operon variants was created by combinatorial assembly of three random RBS libraries for CFP, mRFP1, and GFPmut3b each contained NNNNNN within Shine-Dalgarno region (**Supplementary Table S5**). (B) Five hundred variants were characterized using flow cytometry.
