# Supplementary material for: Efficient search, mapping, and optimization of multi-protein genetic systems in diverse bacteria
Source: Mol Syst Biol. 2014 Jul 1;10(6):731. doi: 10.15252/msb.20134955 (PMC4265053; doi:10.15252/msb.20134955)
Supplement: Supplementary file 3 — Supplementary Figure S3 [file msb0010-0731-sd3.pdf]

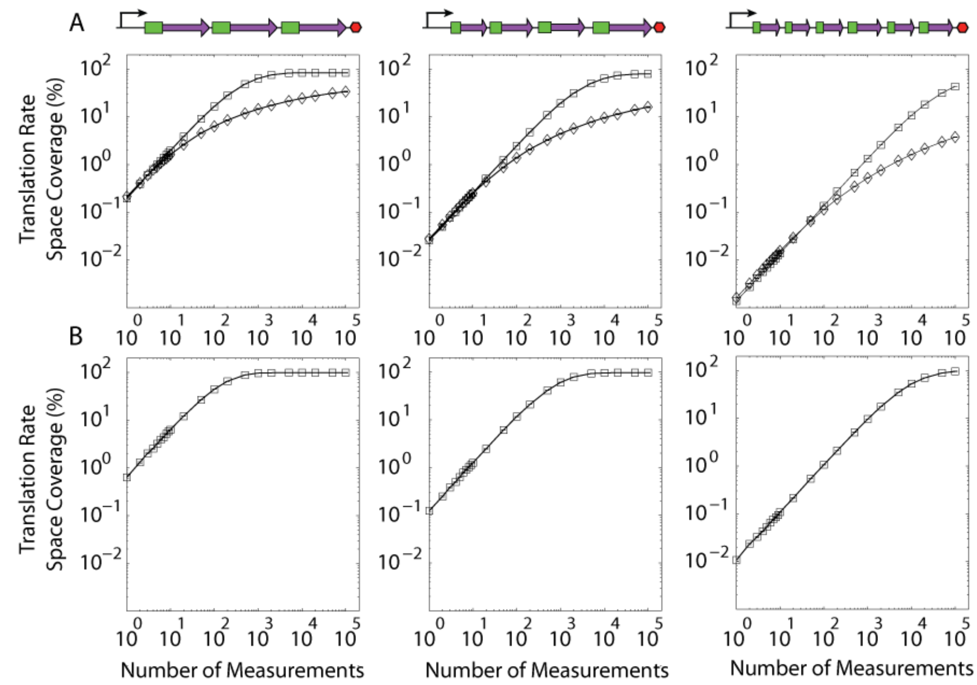

Supplementary Figure S3: Estimating the number of measurements to find optimal protein expression levels in multi-protein genetic systems using random or optimized RBS libraries (A) The number of measurements needed to search for optimal protein expression levels of a genetic system (CrtE, CrtB, CrtI, IspA, Idi, and Dxs) were determined using either optimized RBS libraries (squares) or random RBS libraries (diamonds). First, the entire translation rate space between 1 to 100,000 au was targeted. Random (**Supplementary Table S15**) and optimized (**Supplementary Table S16**) RBS libraries were created and their abilities to cover the translation rate space were compared for three metabolic pathways with (left) three, (middle) four, or (right) six proteins. In all cases, optimized RBS libraries improve coverage and reduce the number of measurements. (B) Zoom mode was employed to design optimized RBS libraries to target a narrow translation rate range between 30,000 and 300,000 au (**Supplementary Table S17**). High coverage of the targeted translation space were achieved using a small number of measurements.
