# Supplementary material for: Efficient search, mapping, and optimization of multi-protein genetic systems in diverse bacteria
Source: Mol Syst Biol. 2014 Jul 1;10(6):731. doi: 10.15252/msb.20134955 (PMC4265053; doi:10.15252/msb.20134955)
Supplement: Supplementary file 4 — Supplementary Figure S4 [file msb0010-0731-sd4.pdf]

$$\text{IPP} + idi \xrightleftharpoons[k_{-1}]{k_1} \text{CM1} \xrightleftharpoons[k_{-2}]{k_2} \text{DMAPP} + idi$$

$$\text{DMAPP} + \text{IPP} + ispA \xrightleftharpoons[k_{-3}]{k_3} \text{CM2} + \text{IPP} \xrightleftharpoons[k_{-4}]{k_4} \text{CM3} \xrightleftharpoons[k_{-5}]{k_5} \text{GPP} + ispA$$

$$\text{GPP} + \text{IPP} + ispA \xrightleftharpoons[k_{-6}]{k_6} \text{CM4} + \text{IPP} \xrightleftharpoons[k_{-7}]{k_7} \text{CM5} \xrightleftharpoons[k_{-8}]{k_8} \text{FPP} + ispA$$

$$\text{GPP} + \text{IPP} + CrtE \xrightleftharpoons[k_{-9}]{k_9} \text{CM6} + \text{IPP} \xrightleftharpoons[k_{-10}]{k_{10}} \text{CM7} \xrightleftharpoons[k_{-11}]{k_{11}} \text{FPP} + CrtE$$

$$\text{FPP} + \text{IPP} + CrtE \xrightleftharpoons[k_{-12}]{k_{12}} \text{CM8} + \text{IPP} \xrightleftharpoons[k_{-13}]{k_{13}} \text{CM9} \xrightleftharpoons[k_{-14}]{k_{14}} \text{GGPP} + CrtE$$

$$2\ \text{GGPP} + CrtB \xrightleftharpoons[k_{-15}]{k_{15}} \text{CM10} \xrightleftharpoons[k_{-16}]{k_{16}} \text{Prephytoene diphosphate} + CrtB$$

$$\text{Prephytoene diphosphate} + CrtB \xrightleftharpoons[k_{-17}]{k_{17}} \text{CM11} \xrightleftharpoons[k_{-18}]{k_{18}} \text{Phytoene} + CrtB$$

$$\text{Phytoene} + CrtI \xrightleftharpoons[k_{-19}]{k_{19}} \text{CM12} \xrightleftharpoons[k_{-20}]{k_{20}} \text{Phytofluene} + CrtI$$

$$\text{Phytofluene} + CrtI \xrightleftharpoons[k_{-21}]{k_{21}} \text{CM13} \xrightleftharpoons[k_{-22}]{k_{22}} \zeta\text{–carotene} + CrtI$$

$$\zeta\text{–carotene} + CrtI \xrightleftharpoons[k_{-23}]{k_{23}} \text{CM14} \xrightleftharpoons[k_{-24}]{k_{24}} \text{Neurosporene} + CrtI$$

Supplementary Figure S4: The proposed elementary reaction model for the Carotenoid biosynthesis pathway. CM1 to CM14 indicates the intermediate enzyme-metabolite complexes. IPP, isopentenyl diphosphate; DMAPP, dimethylallyl diphosphate; GPP, geranyl diphosphate; FPP, farnesyl diphosphate; GGPP, geranyl geranyl diphosphate. Cofactors are omitted.
