# Supplementary material for: Efficient search, mapping, and optimization of multi-protein genetic systems in diverse bacteria
Source: Mol Syst Biol. 2014 Jul 1;10(6):731. doi: 10.15252/msb.20134955 (PMC4265053; doi:10.15252/msb.20134955)
Supplement: Supplementary file 5 — Supplementary Figure S5 [file msb0010-0731-sd5.pdf]

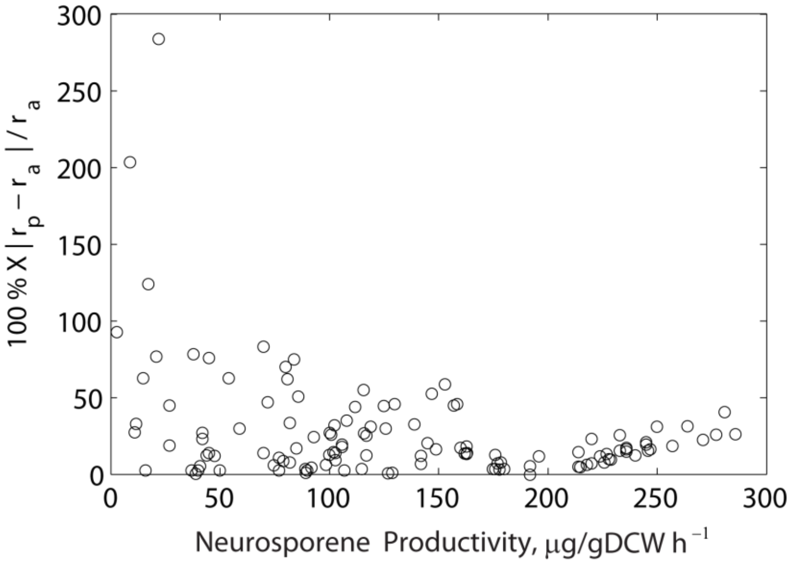

Supplementary Figure S5: The kinetic model's error distribution for neurosporene content predictions. The model's predictions have higher accuracy at higher neurosporene production rate. r_p is the predicted pathway productivity. r_a is the actual, measured pathway productivity. The average error of prediction for all characterized pathways is 32%. There are two outliers responsible for the most error.
