# Supplementary material for: Efficient search, mapping, and optimization of multi-protein genetic systems in diverse bacteria
Source: Mol Syst Biol. 2014 Jul 1;10(6):731. doi: 10.15252/msb.20134955 (PMC4265053; doi:10.15252/msb.20134955)
Supplement: Supplementary file 6 — Supplementary Figure S6 [file msb0010-0731-sd6.pdf]

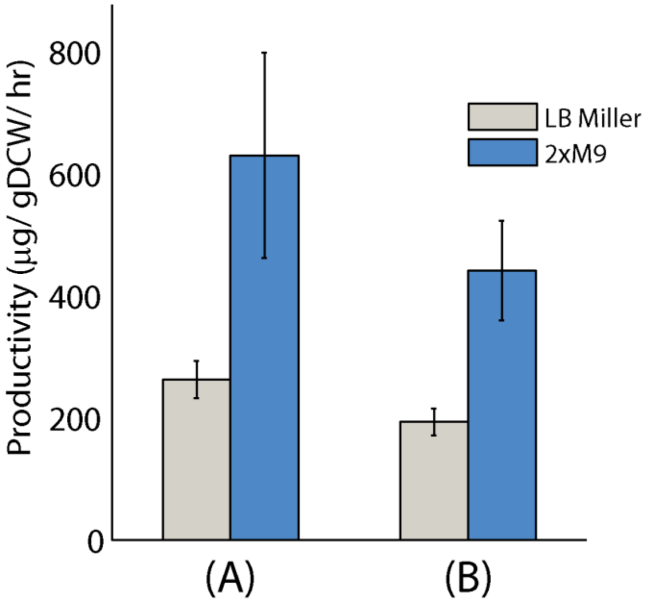

Supplementary Figure S6: Neurosporene content measurements using optimized growth conditions. The Carotenoid pathway variant with the highest neurosporene content was cultivated with high aeration during exponential growth phase for either 7 hrs in LB miller media or 10 hrs in 2xM9 media supplemented with 0.4% glucose. Their neurosporene productivities were measured by hot acetone extraction and dry cell weight measurements. Ten milliliter of each culture was heated for 48 hrs at 60 ˚C and the dry cell weights were measured (A) immediately after baking; or (B) after allowing the samples to equilibrate at room temperature for 4 hrs. The difference in dry cell weight measurements yielded an average difference of 27% in dry cell weight, which resulted in a 28% change in the neurosporene productivity. All carotenoid content and pathway productivity measurements were obtained using the (B) approach, allowing samples to equilibrate after heating.
