# Supplementary material for: Efficient search, mapping, and optimization of multi-protein genetic systems in diverse bacteria
Source: Mol Syst Biol. 2014 Jul 1;10(6):731. doi: 10.15252/msb.20134955 (PMC4265053; doi:10.15252/msb.20134955)
Supplement: Supplementary file 7 — Supplementary Figure S7 [file msb0010-0731-sd7.pdf]

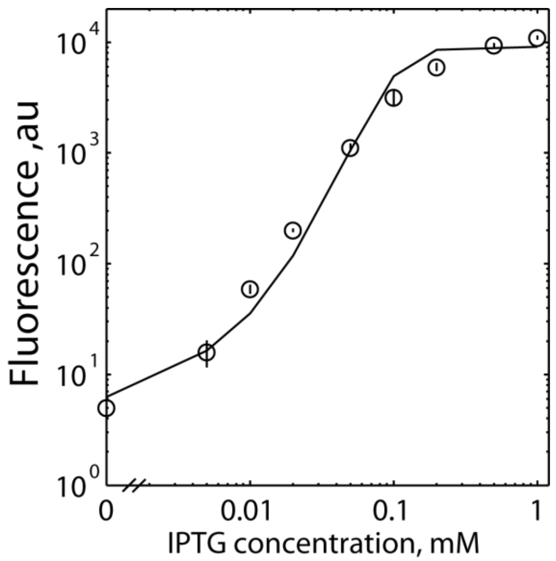

Supplementary Figure S7: Input-output transfer function of P_{lacO1} promoter. A ColE1 based plasmid was constructed to express *mRF1* fluorescence reporter under control of P_{lacO1} promoter, and transformed into the plasmid cured *E. coli* Echw2f. IPTG concentration was varied from 0mM to 1mM, and expression of *mRFP1* fluorescence reporter from P_{lacO1} promoter was measured by flow cytometry during exponential phase in supplemented M9 media. A model of the P_{lacO1} promoter's response to induction was constructed by fitting the experimental data to a hill equation model (Kuhlman et al, 2007), which is $r\,(au) = \frac{9100}{1+\frac{1450}{\left(1+\frac{[IPTG(mM)]}{0.0184}\right)^2}}$.
