# Supplementary material for: Efficient search, mapping, and optimization of multi-protein genetic systems in diverse bacteria
Source: Mol Syst Biol. 2014 Jul 1;10(6):731. doi: 10.15252/msb.20134955 (PMC4265053; doi:10.15252/msb.20134955)
Supplement: Supplementary file 8 — Supplementary Figure S8 [file msb0010-0731-sd8.pdf]

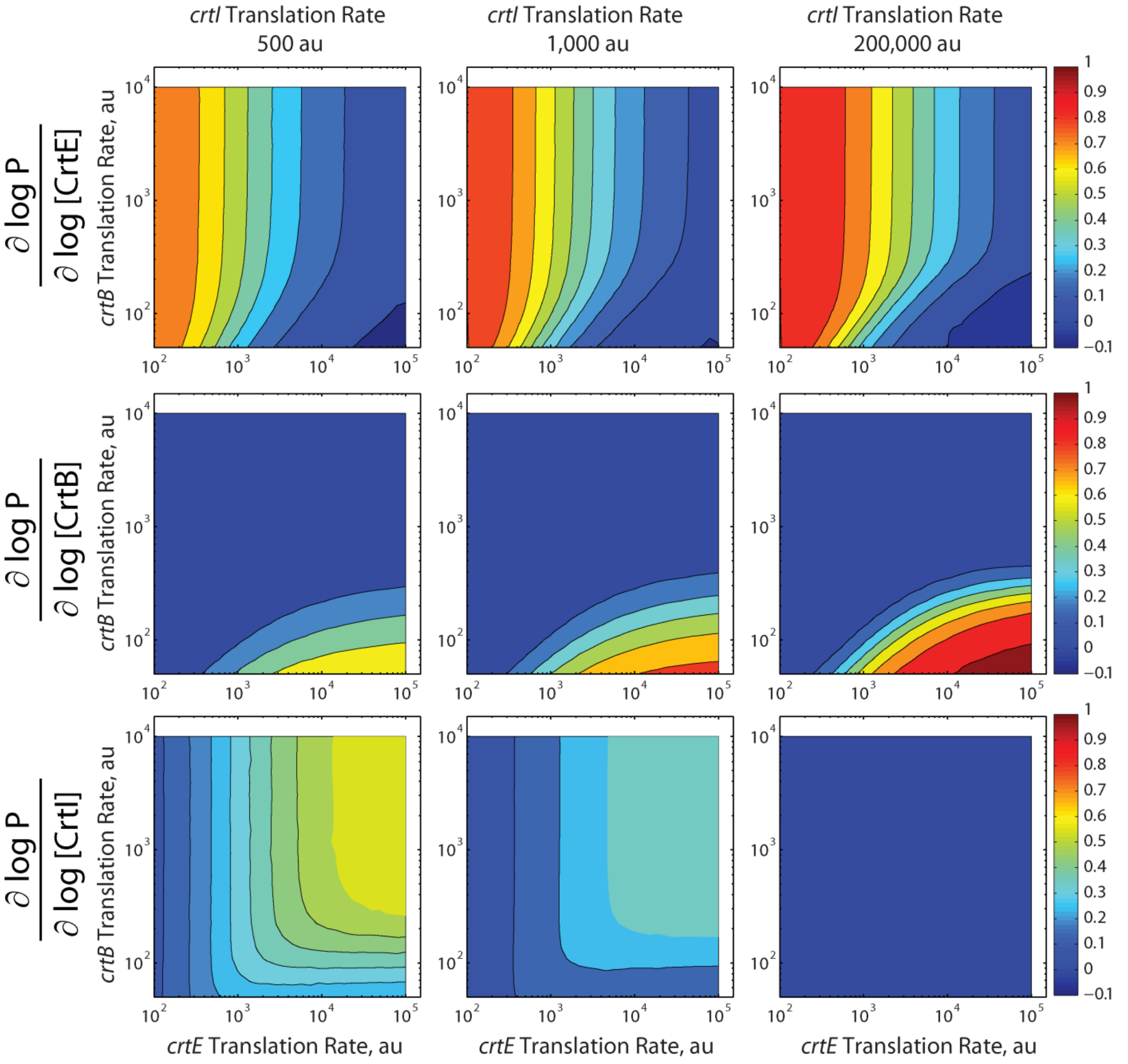

Supplementary Figure 38: Using the kinetic model predictions, flux control coefficients for the enzymes *crtE*, *crtB*, and *crtI* in the neurosporene biosynthesis pathway are evaluated across the 3-dimensional translation rate space. Numerical finite differences are utilized to calculate the partial derivatives at 50x50x50 logarithmically distributed grid points. Contour maps show flux control coefficient values. High FCCs indicate where increasing an enzyme's expression will increase pathway's productivity, while low FCCs show regions where increasing expression does not lead to a significant improvement in productivity. Negative FCCs show regions where excess enzyme expression lowers the pathway's productivity, due to metabolite sequestration.
