# Supplementary material for: Efficient search, mapping, and optimization of multi-protein genetic systems in diverse bacteria
Source: Mol Syst Biol. 2014 Jul 1;10(6):731. doi: 10.15252/msb.20134955 (PMC4265053; doi:10.15252/msb.20134955)
Supplement: Supplementary file 9 — Supplementary Figure S9 [file msb0010-0731-sd9.pdf]

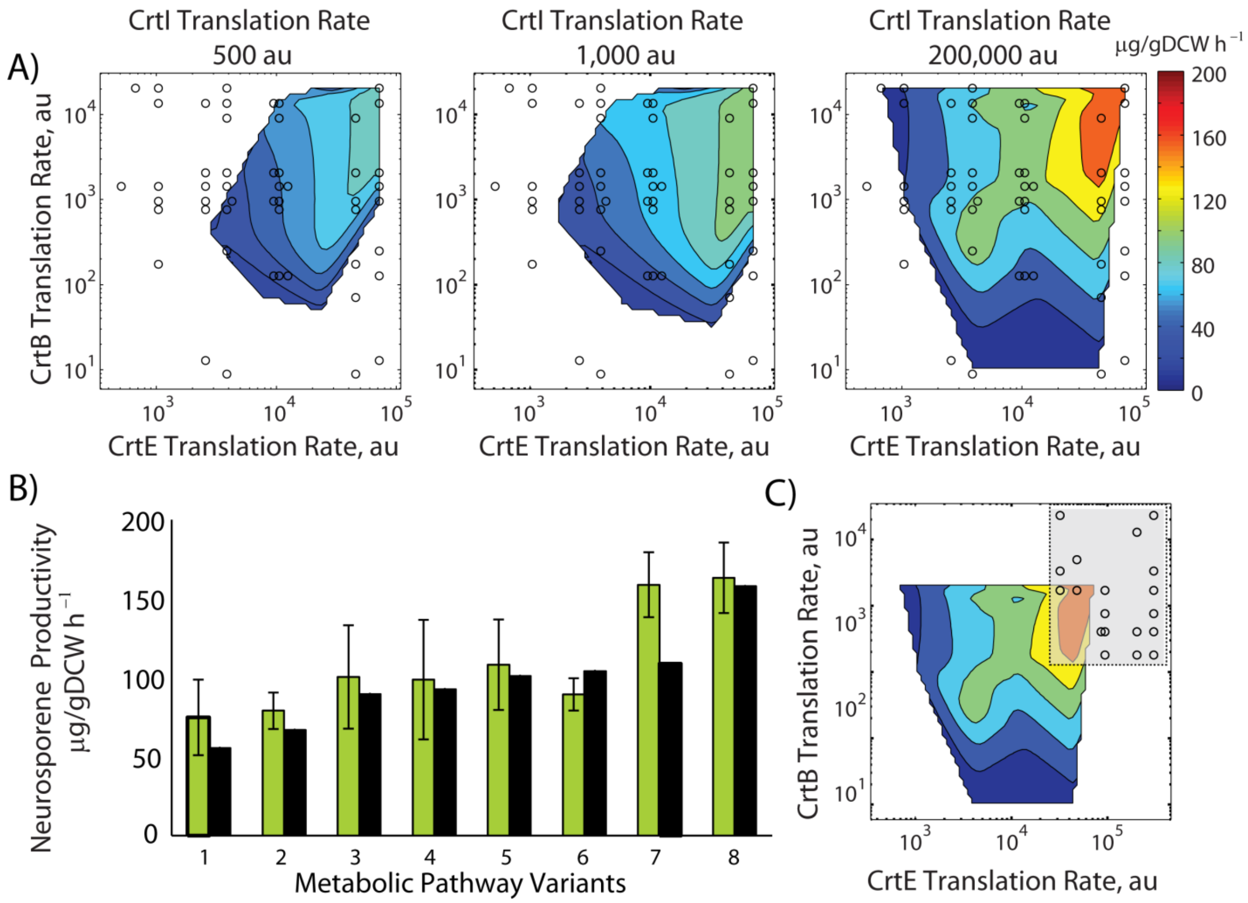

Supplementary Figure S9: Using computational geometry to search for optimal translation rates regions. (A) The CrtEBI expression space is decomposed into Voronoi polygons, and the pathway productivities within the convex hull of the space are computed by interpolation. The white areas are outside the convex hull. (B) 8 out of 19 carotenoid pathway variants from the test data-set (Figure 4E) have *crtEBI* translation rates within the convex hull. Their measured productivities (green) are compared to productivities determined according to geometric interpolation (black). (C) *Zoom* mode can be similarly applied to non-mechanistic models to identify translation rate regions with improved performances.
