# Supplementary material for: Efficient search, mapping, and optimization of multi-protein genetic systems in diverse bacteria
Source: Mol Syst Biol. 2014 Jul 1;10(6):731. doi: 10.15252/msb.20134955 (PMC4265053; doi:10.15252/msb.20134955)
Supplement: Supplementary file 10 — Supplementary Figure S10 [file msb0010-0731-sd10.pdf]

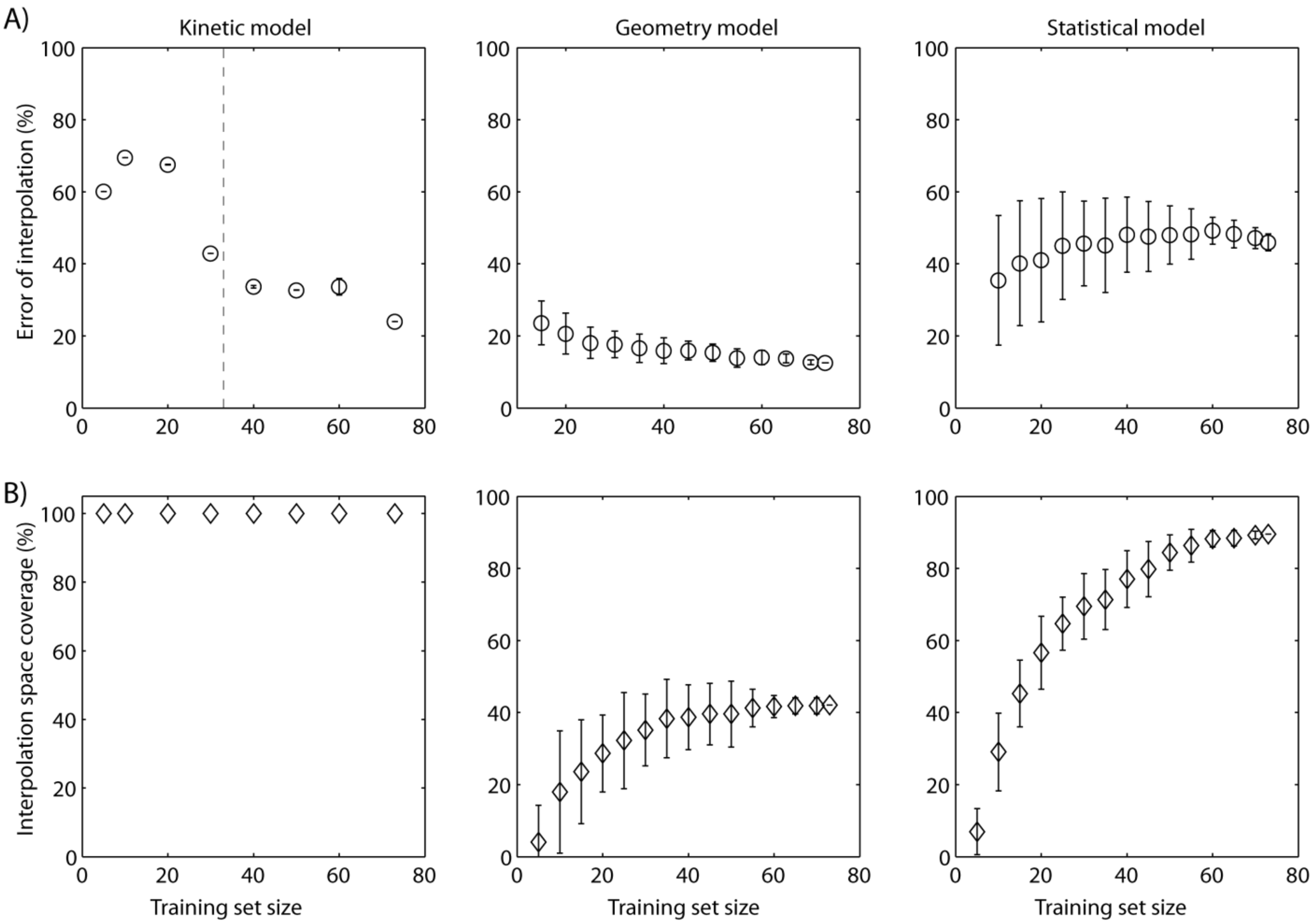


**Supplementary Figure S10:** Effect of training set size on model predictions. (A) Error of interpolation (B) percentage of the test set pathway variants that could be evaluated by the models. To have a reliable prediction from kinetic model, the training set size should be larger than the number of unknown parameters in the model (dashed line).
