# Supplementary material for: Efficient search, mapping, and optimization of multi-protein genetic systems in diverse bacteria
Source: Mol Syst Biol. 2014 Jul 1;10(6):731. doi: 10.15252/msb.20134955 (PMC4265053; doi:10.15252/msb.20134955)
Supplement: Supplementary file 11 — Supplementary Figure S11 [file msb0010-0731-sd11.pdf]

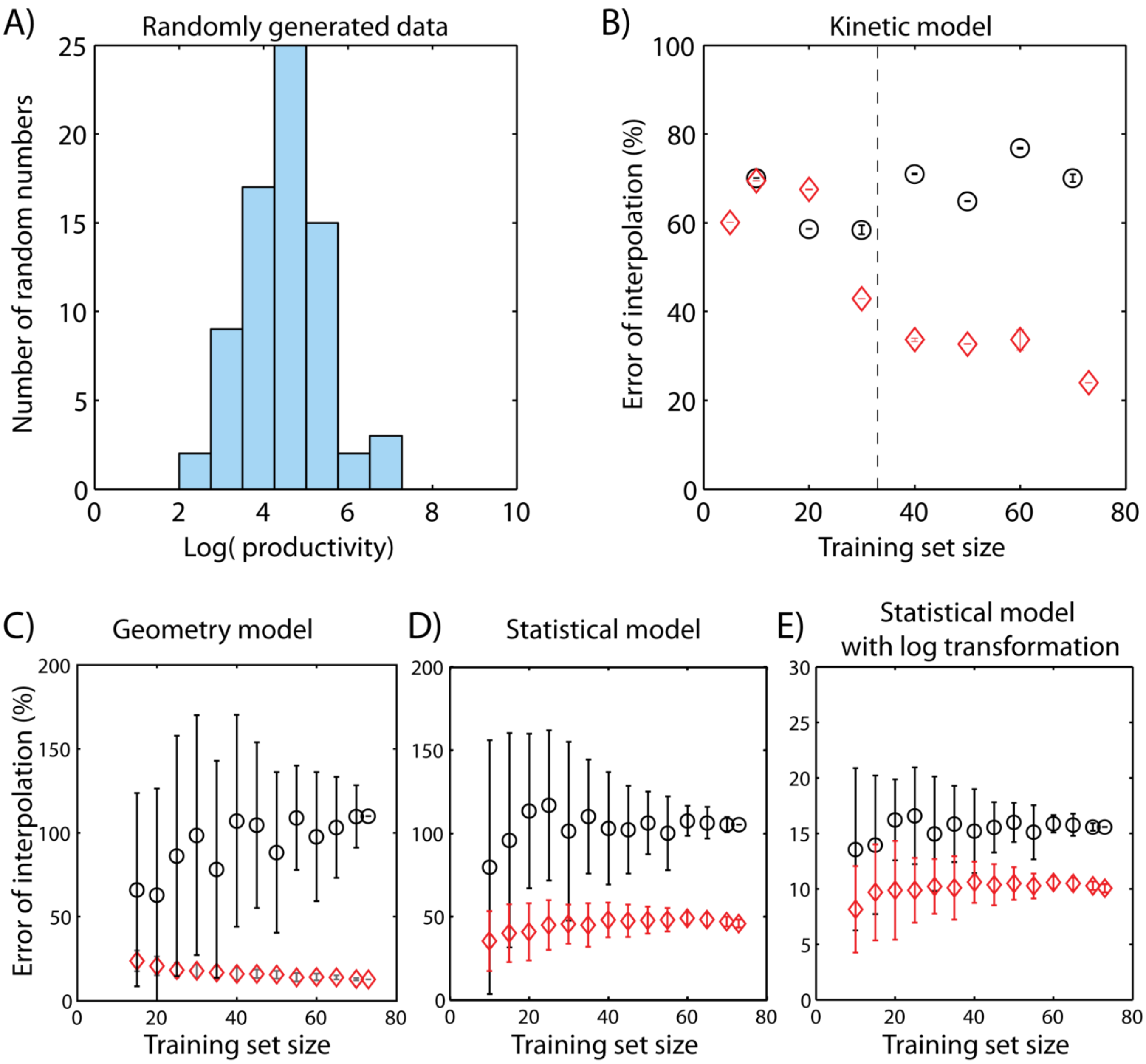

Supplementary Figure S11: Null-hypothesis rejection test. (A) The distribution of the randomly generated productivities, which are used for training. Error of prediction for (B) kinetic (C) Geometry (D) statistical models trained with actual measurement training set (diamond) or randomly generated training set (circle). (E) The model failed to reject the null hypothesis while the other models successfully falsified the null hypothesis.
