# Supplementary material for: Efficient search, mapping, and optimization of multi-protein genetic systems in diverse bacteria
Source: Mol Syst Biol. 2014 Jul 1;10(6):731. doi: 10.15252/msb.20134955 (PMC4265053; doi:10.15252/msb.20134955)
Supplement: Supplementary file 30 — Supplementary Methods [file msb0010-0731-sd30.pdf]

Supplementary Methods

1. Calculating the number of MAGE cycles to insert optimized RBS libraries into a genome
2. Monte-Carlo estimation to determine the minimal number of measurements using *Search* or *Zoom* modes
3. An elementary mass action kinetic model of the carotenoid biosynthesis pathway's reactions
4. Using Computational geometry for building a non-mechanistic model of the Carotenoid pathway
5. Using statistical model for creating a non-mechanistic model of the Carotenoid pathway
6. Performance of kinetic, geometry, and statistical modeling for building a SEAMAP

Supplementary Method 1: The expected number of MAGE cycles to insert optimized RBS libraries into a genome

A general formula is derived for determining the number of MAGE mutagenesis cycles N needed to introduce the optimized RBS libraries into K targeted protein coding sequences within the genome. Multiplexed genome engineering (MAGE) uses mutagenic oligonucleotides and oligo-mediated allelic recombination to incorporate targeted mutations into an engineered host strain.

The efficiency of incorporation is determined by the number of mutagenesis cycles, and depends on the oligonucleotide's allelic replacement efficiency. Several factors affect the allelic replacement efficiency (AR). First, targeting the lagging strand of the genome is 10- to 100-fold more efficient than the leading strand(Ellis et al, 2001). Second, chemically modified oligonucleotides improve AR by increasing the oligonucleotide's intracellular stability and preventing the mismatch DNA repair system (*mutS*) from repairing mutations. AR is generally improved by knocking out *mutS* completely(Costantino). Third, the kinetics of oligonucleotide hybridization are important as there is a 1-second window for allelic recombination(Wang et al, 2012). Inhibitory secondary structures at the 5' and 3' oligonucleotide ends prevent hybridization.

We design oligonucleotides with 5’ phosphorothioate modifications, internal 2' fluoro bases, and minimal 5' and 3' secondary structures to have allelic replacement efficiencies up to 25% in a *mutS* knockout and up to 6% in a *mutS+* strain. Increasing the number of consecutive mutations M in a *mutS* knockout will reduce allelic replacement efficiency according to the mismatch identity(Wang et al, 2009; Wang et al, 2011), following the empirical relationship $AR_{K=1} = 0.26e^{-0.135*(M-1)}$ as determined by Wang et. al(Wang et al, 2009).

The chance of incorporating a single RBS variant into a single site within the genome in one mutagenesis cycle is a Bernoulli trial with probability $AR_{K=1}$. The chance of incorporation increases with additional mutagenesis cycles according to a cumulative geometric distribution, where the number of cycles N determines the total fraction of mutated genome F according to:

$$F_1 = AR_{K=1} + (1 - AR_{K=1})AR_{locus} + (1 - AR_{K=2})^2 AR_{K=1} + \cdots + (1 - AR_{K=1})^{N-1} AR_{K=1}$$

Simplification of this equation leads to:

$$\boldsymbol{F_1 = 1 - (1 - AR_{K=1})^N}$$

where $(1 - AR_{K=1})^N$ is the probability of having a genome remain unmodified after N cycles. For example, the introduction of the stop codon in *lacI* (a C --> T replacement) required only 3 mutagenesis rounds to modify 50% of the genome population (an observed $AR_{K=1}$ of 20%). Introduction of the RBS library into *lacZ* required twenty rounds to modify over 90% of the genome population.

The targeting of multiple RBS sequences and protein coding sequences are largely independent if distantly located (>500 kbp), though the incorporation of mutations at nearby genes may be correlated(Wang & Church, 2011; Wang et al, 2012). Assuming independence and a constant overall oligonucleotide concentration, the fraction of mutated genomes will decrease as additional genes are targeted, according to the relationship:

$$\boldsymbol{F_K = [1 - (1 - AR_{K=1}/K)^N]^K}$$

where the chance of incorporating mutations into two sites is simply $F_1 \times F_1$. The number of mutagenesis cycles N to ensure at least F fraction of the genome has been modified is then:

$$N = \frac{log\left(1 - F^{\frac{1}{K}}\right)}{\log\left(1 - \frac{AR_{K=1}}{K}\right)}$$

When designing mutagenic oligonucleotides, we select up to 7 consecutive mutation variants to ensure a sufficiently high allelic replacement efficiency to maximize genome modification and minimize the number of cycles. According to this design specification, 50% of the genome population will contain at least one modification in each of its K targets with $N = -20K\, log_{10}\left(1 - 0.5^{1}\right.$K cycles. To reduce sequencing costs and increase the fraction of modified genome to 90%, the number of mutagenesis cycles is increased to $N = -20K\, log_{10}\left(1 - 0.9^{1/K}\right)$. Increasing the number of targets while maintaining reasonably low mutagenesis cycles requires the use of coselective MAGE (Wang et al, 2012).

Supplementary Method 2: Monte-Carlo estimation to determine the minimal number of measurements

Monte Carlo sampling could estimate the number of measurements for a library of multi-protein genetic systems that will be sufficient to explore its N-dimensional translation rate space. For each protein in the genetic system, an RBS library is used to vary its translation rate and expression level. The translation rates of RBS sequences will depend on the protein coding sequences in the genetic system. Optimized RBS libraries are designed by the RBS Library Calculator in *Search Mode* to uniformly vary the translation rates of the specified coding sequences between 1 and 100,000 au with a medium search resolution (0.30), yielding libraries with 16 variants. Random RBS libraries contained 6 random nucleotides within their Shine-Dalgarno sequences (ie: NNNNNN or NNNGGANNN) or 9 degenerate consensus nucleotides (DDRRRRRDDDD), creating libraries with 4096, or 23328 variants. In all cases, the translation rates of the RBS library variants are predicted using the RBS Calculator v1.1. Each RBS library presents a pool of choices. Library construction is assumed to generate a homogeneous mixture of RBS variants where all potential choices have equal weight.

For each measurement simulation, one RBS variant is randomly selected from each of the N RBS library pools. The measurement's position in the N-dimensional translation rate space is determined, and its corresponding bin is classified as covered. The number of choices increases combinatorially as the number of proteins in the genetic system increases. Random selection is repeated for M measurements, and the total coverage of the N-dimensional translation rate space is calculated by extension of Equation (4). This process is repeated in 100 independent runs to determine the average and standard deviation of coverage. Monte Carlo sampling is performed identically for optimized or random RBS libraries, and for genetic systems with 2 or more proteins.

Supplementary Method 3: An elementary mass action kinetic model of the carotenoid biosynthesis pathway's reactions

The development of the kinetic model has three parts. First, we derive a mechanistic, mass action model of the pathway to relate changes in enzyme expression level to changes in pathway productivity. Second, we carry out model reduction and dimensional analysis to determine a pathway's productivity in comparison to a reference pathway variant. The dimensional analysis eliminates the need to determine the concentrations of the intracellular metabolites or enzymes, while retaining the ability to predict changes in pathway productivities. Third, we perform model identification to determine the unknown variables' best-fit values that reproduce the experimentally measured pathway productivities. Validation of the kinetic model is performed on an additional set of characterized pathway variants.

Part I. Kinetic model formulation We developed a kinetic model of 10 intracellular reactions catalyzed by Idi, IspA, CrtE, CrtB, and CrtI enzymes that includes (i) reversible binding of substrate to enzyme; and (ii) reversible unbinding of product from enzyme. IspA, CrtE, CrtB, and CrtI catalyze multiple reactions. These reactions convert intracellular isopentenyl diphosphate (IPP) and Dimethylallyl diphosphate (DMAPP) to the neurosporene carotenoid. There are a total of 24 metabolites, free enzyme, and enzyme complexes in the kinetic model (**Supplementary Figure S4**).

Using mass action kinetics, we derive 24 ordinary differential equations describing the rates of production and consumption of all chemical species within the reaction network. We assume isochoric conditions. There are accordingly 48 unknown kinetic parameters quantifying the rates of the first and second order reactions involved in forward and reverse reactions. In addition, five mole balances on the amounts of free and bound enzyme are derived. The amounts of free and bound enzyme must sum to a constant amount. The metabolic dynamics of all 73 pathway variants are governed by the same set of kinetic parameter values and mole balances.

All reactions in the kinetic model are potentially reversible. The net increases in species concentrations (C_i) are determined by the forward ($v_{f,j}$) and reverse ($v_{r,j}$) rates, or the net reaction rates V_j^{net}, of the N_i participating reactions that consume or produce metabolite i, according to:

$$\frac{dC_i}{dt} = F_{in}^i + \sum_{j=1}^{N_i} v_{f,j} - v_{r,j} = F_{in}^i + \sum_{j=1}^{N_i} V_j^{net}, \qquad i\text{=1 to 24}$$

The final reaction's net rate determines the pathway's overall neurosporene productivity.

The rates of the forward and reverse reactions are determined by either first order ($k_i[S_j]$ or $k_{-i}[S_j]$) or second order ($k_i[S_j][S_k]$ or $k_{-i}[S_j][S_k]$) mass action kinetics where k_i and k_{-i} are the kinetic parameters for the forward and reverse reactions, respectively. F_{in}^{i} is the input flux for metabolite i which is zero for all metabolites except IPP and DMAPP. $[S_j]$ refers to the concentration of the jth chemical species, which may be a metabolite, a free enzyme, or a bound enzyme. The net reaction rate is the sum of the forward and reverse reaction rates:

$$V_j^{net} = v_{f,j} - v_{r,j}$$

To illustrate, the first reaction describes the binding of IPP to the enzyme *idi*. Its forward and reverse reaction rates are calculated according to:

$$v_{f,1} = k_1[idi][IPP] \text{ and } v_{r,1} = k_{-1}[CM1]$$

where $[idi]$ is the concentration of free *idi* enzyme, $[CM1]$ is the concentration of bound *idi* enzyme, and $[IPP]$ is the concentration of metabolite isopentenyl diphosphate.

Next, we perform a dimensional analysis to transform unknown metabolite and enzyme concentrations into metabolite and enzyme concentration ratios, using a reference pathway variant for comparison. We multiply and divide the forward and reverse reaction rates by the metabolite and enzyme concentrations within the reference pathway variant, which do not alter the calculated answer. We then re-arrange the variables to yield an apparent kinetic parameter, a metabolite concentration ratio, and an enzyme concentration ratio. As a result, the concentration ratios are bounded and appropriately scaled. To illustrate, we carry out this transformation for the first reaction's forward and reverse rates, yielding:

$$v_{f1} = \underbrace{\left(k_1 * [IPP]_{ref} * [idi]_{ref}^{total}\right)}_{apparent\ kinetic\ parameter} * \underbrace{\frac{[IPP]}{[IPP]_{ref}}}_{\substack{metabolite\\ concentration\\ ratio}} * \underbrace{\frac{[idi]^{free}}{[idi]_{ref}^{total}}}_{\substack{enzyme\\ concentration\\ ratio}}$$

$$v_{r1} = \underbrace{\left(k_{-1} * [CM1]_{ref}\right)}_{apparent\ kinetic\ parameter} * \underbrace{\frac{[CM1]}{[CM1]_{ref}}}_{\substack{enzyme\\ concentration\\ ratio}}$$

Importantly, the choice of the reference pathway variant will alter the apparent kinetic parameter values, but it will not alter the solution to the ordinary differential equations; increases in the apparent kinetic parameters are compensated by decreases in the enzyme concentration ratios. We selected pathway variant #53 as the reference pathway variant. The total enzyme concentration ratios for each pathway variant are determined by comparing that pathway variant's translation rates to the translation rates of the reference pathway. As an example, for *crtE*, the equation is:

$$\underbrace{\frac{[CrtE]^{total}}{[CrtE]_{ref}^{total}}}_{\substack{enzyme\\ concentration\\ ratio}} = \underbrace{\frac{translation\ initiation\ rate\ of\ crtE\ in\ a\ pathway\ variant}{translation\ initiation\ rate\ of\ crtE\ in\ the\ reference\ pathway}}_{\substack{translation\ initiation\ rate\\ ratio}}$$

The reference pathway has predicted translation initiation rates of 72268, 20496, and 203462 au for *crtE*, *crtB*, and *crtI*, respectively.

To determine a pathway's metabolite concentrations and productivity, we input the enzymes' concentrations and kinetic parameters into the ordinary differential equations and utilize a stiff numerical integrator (MATLAB's ode23s) to determine a numerical solution. The time period of the numerical solution is the same as the experimental growth conditions; 7 hours post-induction for all pathway productivity predictions shown in Figure 2. The numerical solution provides the time-dependent metabolite concentrations, including the production flux of neurosporene. We denote the neurosporene production flux as r_p. Each pathway variant will have a different neurosporene production flux as a result of the different total enzyme concentrations, controlled by the *crtEBI* translation rates.

We then use the reference pathway to determine how changes in the model's calculated neurosporene production flux will control the pathway's neurosporene productivity. By comparing the ratio in neurosporene production fluxes between reference and non-reference pathway variants, we predict the non-reference pathway's neurosporene productivity according to the equation:

$$\underbrace{\frac{r_{p,i}}{r_{p,ref}}}_{\substack{production\,flux\\ ratio}} = \underbrace{\frac{predicted\ neurosporene\ productivity\ of\ the\ i^{th}\ pathway\ variant}{measured\ neurosporene\ productivity\ of\ the\ reference\ pathway}}_{\substack{pathway\ productivity\\ ratio}}$$

The neurosporene productivity of the reference pathway is 196 µg/gDCW/hour. The reference pathway's neurosporene production flux is calculated using the same kinetic parameters and mole balances as the non-reference pathway variants.

The kinetic parameters control the pathway's metabolic dynamics and its overall productivity; however, the mass action kinetic parameters for each enzyme in the reaction network (idi, ispA, CrtE, CrtB, and CrtI) are not known, and are difficult to measure. We use model identification to determine the best-fit kinetic parameters that reproduced the measured productivities for the 73 characterized pathway variants.

Prior to carrying out model identification, we employed model reduction and dimensional analysis to eliminate non-independent variables within the kinetic model, and to bound all kinetic parameters, metabolite concentrations, and enzyme concentrations. This procedure reduces the overall number of degrees of freedom within the kinetic model, while enabling the optimization procedure to rapidly converge to a single set of parameter values that satisfies all observations.

Part II. Model reduction and dimensional analysis

We use the ensemble modeling approach(Contador et al, 2009; Tran et al, 2008) to reduce and de-dimensionalize the proposed kinetic model and to convert all unknown kinetic parameters and concentrations into bounded variables. This transformation eliminates time-scale variation and enables the model identification process to rapidly converge to best-fit kinetic parameters with low predictive errors.

First, we eliminate non-independent, redundant kinetic parameters by quantifying the net rate of a reaction in terms of a reaction reversibility. A reaction reversibility R_i of 1 indicates that the ith reaction proceeds only in the forward direction; similarly, a R_i of -1 indicates that the reaction proceeds only in the reverse direction, according to the equations:

$$R_i = \frac{v_{f,i} - v_{r,i}}{v_{f,i} + v_{r,i}} = \frac{V_i^{net}}{v_{f,i} + v_{r,i}} = \frac{C}{v_{f,i} + v_{r,i}}$$

where we note how the net reaction rate and reaction reversibility are directly related. Due to carbon balancing under steady-state conditions, the net reaction rate on a per-carbon basis would become a constant C for all reactions. Therefore, for the reference pathway, we assume that its metabolic dynamics have reached a steady-state condition such that the C becomes a constant. We do not assume steady-state conditions for non-reference pathway variants.

By substituting first or second order rate laws into the forward and reverse reaction rates, we can relate the kinetic parameters and metabolite concentrations to the reaction reversibilities, and eliminate an unknown variable. To illustrate, model reduction can express both the forward and reverse reaction rates using fewer unknown variables:

$$v_{f,i} = \frac{1}{2}C\left(1 - \frac{1}{R_i}\right) \text{ and } v_{r,i} = -\frac{1}{2}C\left(1 + \frac{1}{R_i}\right)$$

where the C constant is a known quantity as it is directly related to the pathway's production rate of neurosporene, which is experimentally measured. Consequently, for each pair of reactions in the reference pathway's kinetic model, we eliminate a non-independent variable.

Overall, there are 24 chemical species and 24 elementary reactions in the reaction network. Without model reduction and dimensional analysis, there are 48 unknown kinetic parameters quantifying the forward and reverse rates. Model identification can be performed on the non-reduced model, but it would result in greater variability in best-fit kinetic parameters, longer optimization convergence times, and a requirement for more characterized pathway variants to achieve the same predictive error. Alternatively, applying model reduction reduces the number of unknown variables to 24 unknown reaction reversibilities and 14 unknown bound enzyme concentration ratios, yielding 38 unknown variables. One additional unknown parameter defines the flux ratio between FPP and GPP entering the system, totaling 39 unknown parameters. These 39 unknown parameters are bounded and well-scaled; they vary between -1 and 1, or between 0 and 1.

Previous characterization of the pathway's enzymes enables a further reduction in the number of unknown variables (Bennett et al, 2009; Rohdich et al, 2003; Schomburg, 2009). Bennete et. al. (Bennett et al, 2009) found that 83% of studied metabolites in *E. coli* central metabolism have intracellular concentrations that are much higher than their enzymes' corresponding Km values, indicating that these enzymes operate near their maximum velocity. We accordingly imposed two additional assumptions in the kinetic model to constrain IPP and geranyl diphosphate (GPP) concentrations to be 10 times higher than their Km values in *ispA* and *idi*. We also set the ratio between production rate of isopentenyl diphosphate and dimethylallyl diphosphate to be 6:1 (Rohdich et al, 2003). These constraints simplified the upper portion of the reaction network that governs flux distribution between IPP, GPP, and FPP, and reduced the total number of unknown variables to 33.

Part III. Model Identification to Identify the Values of the Unknown Variables

Model identification is an optimization procedure that compares a model's solution to a data-set of measured observations. A sufficiently large set of accurate observations will allow precise identification of all unknown variables within a model. Here, we have 72 non-reference pathway variants and one reference pathway variant with accurately measured neurosporene productivities, and a kinetic model with 33 unknown variables. The solution to the kinetic model is the neurosporene production fluxes for the 72 non-reference pathway variants and the one reference pathway variant. We then use reference pathway variant's neurosporene production

flux and measured neurosporene productivity to predict the non-reference pathway variants' neurosporene productivities. The quality of the model identification is determined by comparing the model's predicted neurosporene productivities to the experimentally measured productivities. A perfect kinetic model would have equivalent comparisons across all 72 non-reference pathway variants.

We use genetic algorithm optimization to identify the values of the 33 unknown variables that maximizes the quality of the kinetic model across the 72 non-reference pathway variants. We performed ten separate and independent optimization runs to identify the best-fit values to the unknown variables. We used a genetic algorithm population size of 50, and a convergence tolerance of 1% variation. We then selected the set of parameter values with the lowest error. The resulting kinetic model predicts the non-reference pathway variants' neurosporene productivities to within 32% of the measured productivities (**Supplementary Figure S5**). The same kinetic model is able to predict the productivities of the 19 additional pathway variants to within 24%; the measurements of these pathway variants were not used during the model identification process. Taking into account all constraints, we then performed inverse model reduction to determine the 48 kinetic parameter values that define the identified kinetic model (**Supplementary Table S8**).

The 24 non-reduced ordinary differential equations

$$\frac{d[IPP]}{dt} = F_{IPP} + k_{-1}[CM1] - k_1[idi][IPP] + k_{-4}[CM3] - k_4[CM2][IPP] + k_{-7}[CM5] - k_7[CM4][IPP] + k_{-10}[CM7] - k_{10}[CM6][IPP] + k_{-13}[CM9] - k_{13}[CM8][IPP]$$

$$\frac{d[DMAPP]}{dt} = F_{DMAPP} + k_2[CM1] - k_{-2}[idi][DMAPP] - k_3[ispA][DMAPP] + k_{-3}[CM2]$$

$$\frac{d[GPP]}{dt} = k_5[CM3] - (k_{-5} + k_6)[ispA][GPP] + k_{-6}[CM4] - k_9[CrtE][GPP] + k_{-9}[CM6]$$

$$\frac{d[FPP]}{dt} = k_{11}[CM7] - (k_{-11} + k_{12})[CrtE][FPP] + k_{-12}[CM8] + k_8[CM5] - k_{-8}[FPP][ispA]$$

$$\frac{d[GGPP]}{dt} = k_{14}[CM9] - k_{-14}[CrtE][GGPP] - k_{15}[CrtB][GGPP]^2 + k_{-15}[CM10]$$

$$\frac{d[PPPP]}{dt} = k_{16}[CM10] - (k_{-16} + k_{17})[CrtB][PPPP] + k_{-17}[CM11]$$

$$\frac{d[Phytoene]}{dt} = k_{18}[CM11] - k_{-18}[CrtB][Phytoene] - k_{19}[CrtI][Phytoene] + k_{-19}[CM12]$$

$$\frac{d[Phytofluene]}{dt} = k_{20}[CM12] - (k_{-20} + k_{21})[CrtI][Phytofluene] + k_{-21}[CM13]$$

$$\frac{d[\xi - carotene]}{dt} = k_{22}[CM13] - (k_{-22} + k_{23})[CrtI][\xi - carotene] + k_{-23}[CM14]$$

$$\frac{d[Neurosporene]}{dt} = k_{24}[CM14] - k_{-24}[CrtI][Neurosporene]$$

$$\frac{d[CM1]}{dt} = k_1[idi][IPP] - (k_{-1} + k_2)[CM1] + k_{-2}[idi][DMAPP]$$

$$\frac{d[CM2]}{dt} = k_3[ispA][DMAPP] - k_{-3}[CM2] + k_{-4}[CM3] - k_4[CM2][IPP]$$

$$\frac{d[CM3]}{dt} = k_4[CM2][IPP] - (k_{-4} + k_5)[CM3] + k_{-5}[ispA][GPP]$$
$$\frac{d[CM4]}{dt} = k_6[ispA][GPP] - k_{-6}[CM4] + k_{-7}[CM5] - k_7[CM4][IPP]$$
$$\frac{d[CM5]}{dt} = k_7[CM4][IPP] - (k_{-7} + k_8)[CM5] + k_{-8}[ispA][FPP]$$
$$\frac{d[CM6]}{dt} = k_9[CrtE][GPP] - k_{-9}[CM6] + k_{-10}[CM7] - k_{10}[CM6][IPP]$$
$$\frac{d[CM7]}{dt} = k_{10}[CM6][IPP] - (k_{-10} + k_{11})[CM7] + k_{-11}[CrtE][FPP]$$
$$\frac{d[CM8]}{dt} = k_{12}[CrtE][FPP] - k_{-12}[CM8] + k_{-13}[CM9] - k_{13}[CM8][IPP]$$
$$\frac{d[CM9]}{dt} = k_{13}[CM8][IPP] - (k_{-13} + k_{14})[CM9] + k_{-14}[CrtE][GGPP]$$
$$\frac{d[CM10]}{dt} = k_{15}[CrtB][GGPP]^2 - (k_{-15} + k_{16})[CM10] + k_{-16}[\text{PrePhytoene diphosphate}][CrtB]$$
$$\frac{d[CM11]}{dt} = k_{17}[CrtB][\text{PrePhytoene diphosphate}] - (k_{-17} + k_{18})[CM11] + k_{-18}[Phytoene][CrtB]$$
$$\frac{d[CM12]}{dt} = k_{19}[CrtI][Phytoene] - (k_{-19} + k_{20})[CM12] + k_{-20}[Phytofluene][CrtI]$$
$$\frac{d[CM13]}{dt} = k_{21}[CrtI][Phytofluene] - (k_{-21} + k_{22})[CM13] + k_{-22}[\xi - carotene][CrtI]$$
$$\frac{d[CM14]}{dt} = k_{23}[CrtI][\xi - carotene] - (k_{-23} + k_{24})[CM14] + k_{-24}[Neurosporene][CrtI]$$

The mole balances summing together the free and bound forms of the five enzymes

$$[idi] + [CM1] = [idi]_{total}$$
$$[ispA] + [CM2] + [CM3] + [CM4] + [CM5] = [ispA]_{total}$$
$$[CrtE] + [CM6] + [CM7] + [CM8] + [CM9] = [CrtE]_{total}$$
$$[CrtB] + [CM10] + [CM11] = [CrtB]_{total}$$
$$[CrtI] + [CM12] + [CM13] + [CM14] = [CrtI]_{total}$$

Flux model constraints

The metabolic flux of IPP and DMAPP entering the reaction network has a ratio of about 6:1. This is enforced through a model constraint. $F_{IPP} = \frac{6}{7}, F_{DMAPP} = \frac{1}{7}$

Supplementary Method 4: Using Computational geometry for building a non-mechanistic model of the Carotenoid pathway

There are many types of non-mechanistic models that can capture high-dimensional relationships between inputs (translation rates) and outputs (pathway productivities). As an example, we created a non-mechanistic model that uses computational geometry and linear interpolation to calculate a pathway variant's productivity within a translation space region.

The computational geometric model first partitions the translation rate space into small Voronoi polygons (Bobach et al, 2006). The center of each polygon is a vertex in the 4-dimensional space, defined by the predicted translation rates and measured productivities of a pathway variant. There are 73 Voronoi polygons to represent the 73 pathway variants in the initially characterized data-set. The interior volume of the translation rate space is bounded by the 73 polygon's convex hull. The convex hull forms the border of the translation rate regions where interpolation may be used to calculate the pathway productivity of any point within the interior volume.

Next, we employed linear interpolation to calculate the productivity of a pathway whose enzymes have translation rates within the interior volume of the convex hull. The productivity of the pathway is the distance-weighted sum of the productivities from the surrounding centers of Voronoi polygons (**Supplementary Figure S9A**). It is not possible to use linear interpolation to calculate a pathway's productivity when its translation rates exist outside the convex hull; accordingly, these regions are shown as white areas. 56 out of initially characterized 73 pathway variants have translation rates that exist within the convex hull. Linear interpolation is able to reproduce the measured pathway variants' productivities to within 32%. For the 19 additionally characterized pathway variants, only 8 have translation rates within the convex hull and linear interpolation is able to predict these pathways' productivities to within 15% (**Supplementary Figure S9B**).

Supplementary Method 6: Using statistical model for creating a non-mechanistic model of the Carotenoid pathway

Recently Lee et al.(Lee et al, 2013) engineered Violacein pathway in *Saccharomyces cerevisiae* by developing a linear statistical model that predicts synthesis of the pathway's intermediate and final products. The model's coefficient of determination (R^2) for the log(Violacein) prediction was 0.64 and their model only required characterization data for less 3% of their entire combinatorial library (91 separate clones out of 3125 potential variants). The relatively good R^2 and simplicity of the model motivated us to examine the statistical model for constructing a SEAMAP of Neurosporene biosynthesis pathway.

To build a linear statistical framework, an independent categorical state i is defined as any combination of RBS variants for every targeted gene (total number of states=P), and an unknown parameter (β_i) is assigned for each state to include its fitness in the model's predictions (Y). Combinatorial space of characterized pathway variants is mapped by assigning a binary variable ($X_{j,i}$) for present or absent of categorical variant i in characterized pathway variant j (Total number of characterized pathway variants=N). Since the predictions have an arbitrary unit, an extra parameter (t) is added as a linear translation operator. This map could be interpreted as a mathematical transformation from gene expression space to a virtual high-dimensional simplex A with P vertices ($a_1=[\beta_1,0,0,\ldots 0]_{1xP}$, $a_2=[0,\beta_2,0,\ldots 0]_{1xP}$, . . . , $a_P=[0,0,0,\ldots \beta_P]_{1xP}$). The final linear regression model is presented as:

$$\log(Y_j) = t + X_{j,i}\beta_i \qquad i = 1 \cdots P \ , j = 1 \cdots N$$

The common approach to identify the unknown parameters of a linear regression is least-square. However, according to Lee et al., this may result in poor fitting and unfavorite linear transformation. Instead, Exterior Derivative Estimator (EDE) method(Aswani et al, 2011) could

find better values for the P+1 model parameters by learning the intrinsic data constraints(Lee et al, 2013).

We developed a linear statistical model for Neurosporene biosynthesis pathway. According to Lee et al.(Lee et al, 2013) , a relatively low number of pathway variants, between 1% and 2% (around 30 to 60 clones of their potential 3125 pathway variants), was sufficient to train an accurate predictive model for Violacein pathway. Hence, we chose 73 synthetic Neurosporene pathway variants as the training set (around 2% of total combinatorial library) and a set of additional 19 variants for model validation. The training set contained 44 independent RBS variants for CrtE, CrtB, and CrtI, resulted in a final linear regression model with 45 unknown parameters (P=44). We used EDE method to train the model. This category-based model can only provide information about a pathway variant if its RBS variants exist in the training set. One CrtI RBS variant (TIR=312 au) was present in two pathway variants (out of 19 variants) of the test set but did not exist in any of the 73 training variants thus, these data were excluded from error calculation. The average error of productivity prediction for the remaining test variants was 46%.

The proposed statistical model can only perform limited interpolation in discrete intervals of Neurosporene expression-activity space. This prevents the model from estimating the productivity of novel pathway variants (with uncharacterized RBS sequences). As a result, the model fails to identify highly productive pathways; the best computed productivity by the linear model is 145 µg/gDCW/hr while the highest measured productivity among the training set and all characterized variants are 196 and 286 µg/gDCW/hr respectively.

Supplementary Method 7: Performance of kinetic, geometry, and statistical modeling for building a SEAMAP

Biophysical modeling of gene expression(Borujeni et al, 2013; Salis, 2011; Salis et al, 2009) could be coupled with a kinetic, statistical, or geometry modeling to quantitatively link microorganisms' DNA sequences with their phenotypic behaviors. Availability and quality of characterization data, complexity of the pathway, and application of the map affects the competency of the choices. Here, we use Neurosporene biosynthesis pathway to compare these models. We analyze their interpolation and extrapolation predictive powers, the effect of data throughput on their predictions, and their ability to falsify incorrect data acquisition and mathematical transformation.

The first criterion for selecting a model is usually its interpolation and extrapolation power. We evaluated interpolation power of the trained kinetic, geometry, and statistical models by estimating pathway productivity for a set of 19 Neurosporene pathway variants. These models were able to provide information for 100%, 42%, and 89% of the test variants with average errors of 24.8%, 12.5%, and 46% respectively. We then tested their extrapolation power for additional 28 pathway variants with high enzyme expression levels. The kinetic model estimated the productivity for the entire set with 17% error. However, both geometry and statistical models failed to reveal any information about the extrapolation set.

The smallest number of measurements to develop a reliable model is also an essential model criterion. To study the effect of data throughput, we varied the training set size by randomly selecting 5 to 73 pathway variants, retraining the kinetic, geometry, and statistical models, and calculating the average relative error for 19 test variants. Any pathway variant, which existed in spatial gaps of geometry or statistical models, was excluded from their error calculations. With any number of training data, the kinetic model evaluated the behavior of entire test set while geometry model estimated the productivity of the only variants that were inside the convex hull

formed by the training set data (**Supplementary Figure** 10A). The later model locally has a great predictive power and enables smooth and continuous predictions but requires large amount of data to cover a large region in expression-activity space. Unlike kinetic and geometry models, the linear statistical model is a discrete model and, it can only predict the phenotypic behavior of a pathway variant if all its RBS sequences exist as parts of training set pathways. The number of training measurements significantly influences predictions of this model (**Supplementary Figure** 10B).

An incorrect mathematical transformation could hide intrinsic characteristics of data, and falsely show high predictive power for a simplified model. For instance, just by changing error definition from Formula S1 to Formula S2, one can artificially lower prediction error of an identical statistical model from 46% to 10%. Thus, any new model needs to be evaluated for false-positive rejection. Here, as a null hypothesis, we trained the kinetic, geometry, and statistical models with randomly generated data (instead of the actual experimental productivity measurements) and calculated error of prediction for the existing test set. We generated 73 random numbers (**Supplementary Figure** 11A) utilizing a normal distribution random generator (with mean and standard deviation of actual log (productivity) for training set), and trained the proposed models with randomly selected training sets that contained 10 to 73 generated variants. A good model that was trained by the actual measurements should provide small prediction error for the test set (Ea) while training the same model with the randomly generated data should result in a large calculation error (E_R). The E_R /Ea ratio determines the maximum error dynamic range that could be achieved by the model using the training and test sets. We define successful null-hypothesis rejection for a model if its dynamic range is at least 2-fold. When using Formula S1 for defining error, we observed successful null-hypothesis rejection for the kinetic, geometry, and statistical models (**Supplementary Figure** 11BCD). Lee et al.(Borujeni et al, 2013) compared log(measurements) with log(predictions) by a statistical model for estimating Violacein production and achieved relatively high R^2. To study the effect of log transformation on results of Neurosporene statistical model, we used Formula S2 for error calculation of the model. As a result, error of prediction from identical statistical model substantially reduced (10% error). The calculated error for the null hypothesis also dropped to 15.5% and resulted in model failure for rejecting the null-hypothesis (**Supplementary Figure** 11E). Log-transformation is commonly used for linearizing data. However, this mathematical transformation could be misleading by artificially lowering the prediction error for quantities that vary only one or two orders of magnitudes.

$$Error\ 1(\%) = 100 * \sum_{i=1}^{N} abs\left(\frac{Measurement_i - Prediction_i}{Measurement_i}\right) \quad \text{Formula S1}$$

$$Error\ 2(\%) = 100 * \sum_{i=1}^{N} abs\left(\frac{log(Measurement_i) - log(Prediction_i)}{log(Measurement_i)}\right) \quad \text{Formula S2}$$

A summary of strengths and weaknesses of kinetic, geometry, and statistical modeling for building a SEAMAP has been provided in Supplementary Table 18. Success rate of statistical models is a function of data richness; when high-throughput data is available, statistical models might be the simplest (not an accurate way) to develop a SEAMAP. Geometry models perform precise interpolation but require massive characterizations for large pathways. These models could be used locally to calculate a quantity such as flux control coefficients of a pathway variant's enzymes without acquiring information about the entire expression space. Kinetic modeling utilizes the pre-existing biochemistry information (ex. Km of each enzymes and stoichiometry of the involved reactions) and it only requires small number of characterizations to provide information across sequence-expression activity map.

7. References

Aswani A, Bickel P, Tomlin C (2011) Regression on manifolds: Estimation of the exterior derivative. *The Annals of Statistics* **39:** 48-81

Bennett BD, Kimball EH, Gao M, Osterhout R, Van Dien SJ, Rabinowitz JD (2009) Absolute metabolite concentrations and implied enzyme active site occupancy in Escherichia coli. *Nature chemical biology* **5:** 593-599

Bobach T, Hering-Bertram M, Umlauf G (2006) Comparison of Voronoi based scattered data interpolation schemes. In *Proceedings of International Conference on Visualization, Imaging and Image Processing*, pp 342-349.

Borujeni AE, Channarasappa AS, Salis HM (2013) Translation rate is controlled by coupled trade-offs between site accessibility, selective RNA unfolding and sliding at upstream standby sites. *Nucleic acids research***:** gkt1139

Contador CA, Rizk ML, Asenjo JA, Liao JC (2009) Ensemble modeling for strain development of l-lysine-producing< i> Escherichia coli</i>. *Metabolic Engineering* **11:** 221-233

Costantino N Court, DL (2003) Enhanced levels of lambda Red-mediated recombinants in mismatch repair mutants. *Proc Natl Acad Sci USA* **100:** 15748-15753

Ellis HM, Yu D, DiTizio T (2001) High efficiency mutagenesis, repair, and engineering of chromosomal DNA using single-stranded oligonucleotides. *Proceedings of the National Academy of Sciences* **98:** 6742-6746

Kuhlman T, Zhang Z, Saier MH, Hwa T (2007) Combinatorial transcriptional control of the lactose operon of Escherichia coli. *Proceedings of the National Academy of Sciences* **104:** 6043-6048

Lee ME, Aswani A, Han AS, Tomlin CJ, Dueber JE (2013) Expression-level optimization of a multi-enzyme pathway in the absence of a high-throughput assay. *Nucleic acids research* **41:** 10668-10678

Rohdich F, Zepeck F, Adam P, Hecht S, Kaiser J, Laupitz R, Gräwert T, Amslinger S, Eisenreich W, Bacher A (2003) The deoxyxylulose phosphate pathway of isoprenoid biosynthesis: studies on the mechanisms of the reactions catalyzed by IspG and IspH protein. *Proceedings of the National Academy of Sciences* **100:** 1586-1591

Salis HM (2011) The ribosome binding site calculator. *Methods Enzymol* **498:** 19-42

Salis HM, Mirsky EA, Voigt CA (2009) Automated design of synthetic ribosome binding sites to control protein expression. *Nature biotechnology* **27:** 946-950

Schomburg D (2009) A metabolic network described in absolute terms. *Nature chemical biology* **5:** 535-536

Tran LM, Rizk ML, Liao JC (2008) Ensemble modeling of metabolic networks. *Biophysical journal* **95:** 5606-5617

Wang HH, Church GM (2011) Multiplexed genome engineering and genotyping methods applications for synthetic biology and metabolic engineering. *Methods in enzymology* **498:** 409-426

Wang HH, Isaacs FJ, Carr Pa, Sun ZZ, Xu G, Forest CR, Church GM (2009) Programming cells by multiplex genome engineering and accelerated evolution. *Nature* **460:** 894-898

Wang HH, Kim H, Cong L, Jeong J, Bang D, Church GM (2012) Genome-scale promoter engineering by coselection MAGE. *Nature methods* **9:** 591-593

Wang HH, Xu G, Vonner AJ, Church G (2011) Modified bases enable high-efficiency oligonucleotide-mediated allelic replacement via mismatch repair evasion. *Nucleic acids research* **39:** 7336-7347
