# Supplementary material for: Efficient search, mapping, and optimization of multi-protein genetic systems in diverse bacteria
Source: Mol Syst Biol. 2014 Jul 1;10(6):731. doi: 10.15252/msb.20134955 (PMC4265053; doi:10.15252/msb.20134955)
Supplement: Supplementary file 31 — Supplementary Table Legends [file msb0010-0731-sd31.pdf]

Supplementary Table Legends

Supplementary Table S1: Characterization data for all RBS library variants controlling protein expression. The measured fluorescences for optimized RBS library variants controlling expression of mRFP1, sfGFP, LacZ, GFPmut3B, and adhp::GFPmut3B. Translation initiation rates are predicted using RBS Calculator v1.1.

Supplementary Table S2: Strains and plasmids used in the study. This table contains the DNA content of the studied and created plasmids and strains.

Supplementary Table S3: Designed MAGE oligonucleotides for genome engineering. Two chemically modified 90-mer oligonucleotides were synthesized to introduce a stop codon into lacI and an optimized RBS library to uniformly control *lacZ* expression. The 5' phosphothiorate (*) and 2' fluro uracil (/i2FU/) modifications are denoted. LacZ_oligo_m1_v1(**) oligonucleotide was, used as described in Wang et al, to create LacZ mutant. LacZ_restore oligonucleotide was design to recover lacZ activity during Co-selection MAGE.

Supplementary Table S4: The optimized RBS libraries for sampling the translation rate space of the 3-color CFP, mRFP1, and GFPmut3b operon. The RBS Library Calculator in *Search* mode was used to design three 8-variant RBS libraries with a search resolution of 0.35. Translation initiation rates are predicted using RBS Calculator v1.1.

Supplementary Table S5: Random RBS libraries for sampling translation rate space of the designed triple-color operon. The table contains the random RBS libraries controlling expression of CFP, mRFP1, and GFPmut3b, and their predicted translation rates. Translation initiation rates are predicted using RBS Calculator v1.1.

Supplementary Table S6: The optimized RBS libraries for searching the enzyme expression space of the Carotenoid biosynthesis pathway. The RBS Library Calculator in *Search* mode was used to design 16-variant RBS libraries controlling CrtE, CrtB, and CrtI expression. Translation initiation rates are predicted using RBS Calculator v1.1. When multiple in-frame start codons are present, each capable of producing a full length or extended protein, we select the start codon with the highest predicted translation initiation rate. In particular, there is an extra in-frame GTG start codon in the RBS controlling CrtE expression with a calculated translation initiation rate between 445 and 2585 au. There are internal start codons in two RBS variants controlling CrtI expression; their calculated translation initiation rates are 189 au and 558 au. In addition, two of the 73 pathway variants were observed to have single point mutations in the CrtE RBS, and their translation initiation rates were calculated and used instead of the originally designed RBS sequence.

Supplementary Table S7: Characterization data for the 73 Carotenoid pathways generated using *Search* mode. Their translation rate predictions and neurosporene content measurements are shown. Translation initiation rates are predicted using RBS Calculator v1.1. The carotenoid pathways' predicted productivities were determined using either the kinetic model or the computational geometry model.

Supplementary Table S8: The carotenoid pathway's kinetic parameters determined by model identification. The calculated best-fit 48 kinetic parameters for all 24 elementary reactions of the proposed Carotenoid pathway's kinetic model are shown. Sensitivity analysis revealed that a 2-fold increase or decrease in a single parameter increased the prediction's error to 35%. However, a 10-fold increase or decrease in any two parameters (all pair-wise combinations) increased the prediction's error to 46%. In addition, multiplying all kinetic parameters by a positive factor does not alter the model's predictions. Therefore, model identification resulted in identifying best-fit kinetic parameters that are sensitive to 2-parameter variations, but insensitive to 1-parameter or time-scale changes. As a result, the best-fit kinetic parameters are not unique, but are located within a relatively small manifold within the large kinetic parameter space.

Supplementary Table S9: Characterization of 19 additional carotenoid pathway variants to test the kinetic model's predictions. The predicted translation initiation rates and measured carotenoid productivities are shown. Translation initiation rates are predicted using RBS Calculator v1.1. The kinetic model predicts the carotenoid pathway's productivity at selected *crtEBI* translation rates. The pathway's productivities were predicted with an error of 25%. Stars (*) denote where the in-frame GTG start codon was determined to have a higher predicted translation initiation rate than the ATG start codon. Any pathway variants that is outside the convex hull formed the training data cannot be analyzed by the geometry model (NaN).

Supplementary Table S10: The optimized RBS libraries for targeting CrtE, CrtB, and CrtI expression levels using *Zoom* mode. The translation rate ranges were 32,000 to 305,000 au for CrtE, 1800 to 232,000 au for CrtB, and 26,500 to 1,347,000 au for CrtI. Translation initiation rates are predicted using RBS Calculator v1.1.

Supplementary Table S11: Characterization of 28 Carotenoid pathway variants generated using *Zoom* mode. These variants target the high neurosporene content region in the translation rate space of CrtE, CrtB, and CrtI.

Supplementary Table S12: Characterization of pathways with novel input-output transfer functions. Four pathway variants were designed by SEAMAP to have linear-linear or log-linear behaviors.

Supplementary Table S13: The designed RBS library for varying expression of dxs enzymes. The optimized RBS library for altering translation rate of dxs enzyme generated by *Genome Editing* mode of the RBS Library Calculator was shown.

Supplementary Table S14: Characterization of balanced and imbalanced pathways. The measured neurosporene productivities of the characterized balanced and imbalanced pathways at 16 different dxs production rates were shown.

Supplementary Table S15: The random RBS libraries used to calculate the coverages of example 3-, 4-, and 6-protein expression spaces. The random libraries controlling *crtE*, *crtB*, *crtI*, *dxs*, *idi*, *ispA* are shown. The translation rates were predicted by RBS Calculator v1.1.

Supplementary Table S16: The optimized RBS libraries used to calculate the coverages of example 3-, 4-, and 6-protein expression spaces using Search mode. Six optimized RBS libraries were designed to cover the translation rate space of *crtE*, *crtB*, *crtI*, *dxs*, *idi*, and *ispA*. The translation rates were predicted by RBS Calculator v1.1.

Supplementary Table S17: The optimized RBS libraries used to calculate the coverages of example 3-, 4-, and 6-protein expression spaces using *Zoom* mode. Optimized RBS libraries for *crtE*, *crtB*, *crtI*, *dxs*, *idi*, and *ispA* enzymes were designed to cover a narrow translation rate range between 30,000 and 300,000 au. The translation rates were predicted by RBS Calculator v1.1.

Supplementary Table S18: Comparison between kinetic, geometry, and statistical model for building a SEAMAP. Statistical model 1 and model 2 use formula S1 and S2 for error calculation respectively. The Run time is the order of magnitude of time required for evaluating a pathway variant using MATLAB software on windows 7 32-bit, 2 Quad CPU 2.83 GHz. * They could not provide information for internal gaps in expression space.
